# *SLC12A7* promotes malignant phenotypes of gastric cancer cells and is associated with Ezrin-related PI3K/AKT signaling

**DOI:** 10.3389/fcell.2026.1828288

**Published:** 2026-09-15

**Authors:** Zhijian Li, Shuo Li, Tangyi Xiao, Songhe Yu, Tianbiao Zhang, Ying Zhao

**Affiliations:** 1 Department of General Surgery, Shengjing Hospital of China Medical University, Shenyang, China; 2 Department of Biochemistry & Molecular Biology, China Medical University, Shenyang, China

**Keywords:** Ezrin, gastric cancer, PI3K/AKT, SLC12A7, tumor progression

## Abstract

**Background:**

Gastric cancer (GC) remains a major cause of cancer-related mortality, and the molecular drivers underlying GC progression require further clarification. SLC12A7 has been implicated in several malignancies, but its clinical significance and biological function in GC remain incompletely understood.

**Objective:**

To investigate the clinical significance and functional role of SLC12A7 in GC and to explore whether Ezrin-associated PI3K/AKT signaling is involved in SLC12A7-mediated malignant phenotypes.

**Methods:**

TCGA-STAD RNA-seq data, GEO datasets (GSE29272, GSE3438, and GSE66229), and tissue microarrays were integrated to evaluate SLC12A7 expression and its clinicopathological relevance in Gastric cancer. STRING analysis, molecular docking, and immunofluorescence colocalization were used to assess the potential association between SLC12A7 and Ezrin. Functional assays, including siRNA-mediated knockdown, Ezrin re-expression, CCK-8, Transwell, flow cytometry, qRT-PCR, and Western blotting, were performed to examine proliferation, migration, invasion, cell-cycle distribution, apoptosis, EMT-associated markers, and PI3K/AKT signaling in GC cells.

**Results:**

SLC12A7 was upregulated in GC tissues and cell lines and was associated with selected clinicopathological characteristics and shorter survival. *SLC12A7* knockdown suppressed GC-cell proliferation, migration, and invasion, induced G2/M cell-cycle arrest and apoptosis, reduced PI3K/AKT phosphorylation, increased E-cadherin, cleaved caspase-3, and cleaved caspase-9, and decreased vimentin, cyclin A2, and CDK1. *SLC12A7* expression was positively correlated with Ezrin expression, and partial colocalization of *SLC12A7* and Ezrin was observed in GC cells. Ezrin re-expression partially restored PI3K/AKT phosphorylation and attenuated the inhibitory effects caused by *SLC12A7* knockdown.

**Conclusion:**

*SLC12A7* may promote malignant phenotypes in GC cells through an Ezrin-associated PI3K/AKT signaling mechanism, supporting its potential value as a biomarker and therapeutic target in GC.

## Introduction

Gastric cancer (GC) is the fifth most common malignant tumor worldwide and the third most lethal, causing severe challenges to human health (Sung et al., 2021). According to the analysis of epidemic situation of malignant tumors in China in 2022, there is an estimated 359,000 new GC cases and 260,000 deaths. Since most GC patients have no specific symptoms in early stages, most cases are diagnosed in the middle and late stages of the disease, mostly accompanied by metastasis, resulting in poor clinical treatment outcomes and shortened survival. Currently, targeting therapy, immune checkpoint inhibitors (ICIs) and chemotherapy consists of the major therapies after cytoreductive surgery (Wang et al., 2021), among which targeting therapy is the most promising strategy. The development and application of the targeting pharmaceutics bring novel clinical treatment methods for GC patients in middle and late stages and improve the prognosis (Hsu and Raufi, 2021). Therefore, developing new effective targets for pharmaceutical treatment in GC is of great significance for both GC patients and clinical research.

The solute-carrier family 12 (SLC12) proteins are electroneutral potassium–chloride co-transporters that reside in the plasma membrane. These cotransporters are also named KCCs, and they play an important role in regulating cell volume, chloride concentration in neurons, transepithelial ion movement, as well as blood pressure (Merc et al., 2000). *SLC12A7* is a member of the *SLC12* gene family, located on chromosome 5p15, encoding transmembrane protein KCC4, which is responsible for modulating cell volume through transmembrane transportation of potassium and chlorine in a variety cell types (Merc et al., 2000; Mount et al., 1999). SLC12 family members share a high degree of amino acid sequence homology. Each subtype has distinct physiological functions and expression patterns in the human body, and at least three subtypes have been reported to be associated with cancer growth, migration, and invasion (Garneau et al., 2019). Brown et al., (2018) had proved that *SLC12A7* has a significant effect in promoting tumor cell invasion *in vitro*, they found that amplification of *SLC12A7* may promote tumor cell migration and invasion in adrenal cortical carcinoma via regulating cell membrane components and disrupting osmotic signaling. Hsu et al. inhibited the invasion of breast cancer cells by silencing *SLC12A7* gene, but the inhibition had no effect on cell viability or proliferation (Hsu et al., 2007a). It has also been demonstrated that *SLC12A7* is significantly overexpressed in both cervical and ovarian cancers (Hsu et al., 2007b; Chen et al., 2009), its overexpression is associated with local tumor infiltration, lymph node metastasis, and poor prognosis in the aforementioned cancers. In addition, it was found that the *SLC12A7* gene plays a certain regulatory role in the occurrence and development of various tumors (Chen et al., 2010). However, the molecular mechanism of *SLC12A7* functioning in GC is not yet clear. Therefore, clarifying the impact of *SLC12A7* on the malignant biological behavior of GC and its molecular mechanism of action during its occurrence and development is of great significance for the diagnosis, treatment, and prognosis evaluation of GC.

Ezrin is the member of Ezrin/radixin/moesin family, encoded by *EZR* gene. As a cell membrane-cytoskeleton junction protein, Ezrin functions as a bridge between actin cytoskeleton and membrane associated proteins. It can modulate cellular signaling pathways related to different biological processes, promote various signal transduction processes in the process of tumorigenesis, and mediate multiple basic functions through interactions with various growth factor receptors and adhesion molecules (Li et al., 2021). Ezrin dysfunction is associated with the progression of several diseases, and is essential for epithelial-mesenchymal transition (EMT). EMT process allows cancer cells to migrate from their primary sites, enhances their metastatic ability and invasiveness, regulates the function of EMT-related transcription factors such as Snail and Twist, and activates signaling pathways that further promote EMT processes, such as NF-κB pathway (Kalluri and Weinberg, 2009; Kong et al., 2016; Li et al., 2017). Previous studies have shown that the high expression of Ezrin is related to the survival rate of breast cancer, esophageal cancer, lung cancer and gastric cancer (Li N. et al., 2019; Ling et al., 2010; Zhang et al., 2012; Bal et al., 2007). Research has pointed out that Ezrin protein is upregulated in GC and is associated with tumor size, tumor location, lymph node infiltration and metastasis, as well as shortened survival in stages I, II, and III (Li et al., 2011). Other studies have shown that Ezrin is associated with the p85 subunit and can activate the phosphatidylinositol 3-kinase (PI3K)/protein kinase B (PKB, or AKT) signaling pathway, thereby promoting tumor occurrence and metastasis (Song et al., 2020). Therefore, Ezrin may serve as a potential target to influence the progression of GC via regulating the PI3K/AKT signaling pathway.

Previous studies on *SLC12A7*, Ezrin, and gastric cancer metastasis models provide relevant indirect support for the potential involvement of the *SLC12A7*–Ezrin interaction in tumor progression and metastasis. Weng et al., (2013) reported that the glycosylation of *SLC12A7* is critical for its membrane localization and function. In a mouse xenograft model, deglycosylated *SLC12A7* exhibited reduced tumor formation and lung colonization, suggesting that functional *SLC12A7* may contribute to tumor growth and metastatic dissemination. In gastric cancer, Ezrin-related mechanisms have also been implicated *in vivo* metastasis. Chen et al., (2009) reported that *SLC12A7* co-localized with the cell membrane cytoskeleton Ezrin on the plate-like pseudopodia of cancer cells, and enhanced cancer migration and invasion. Zhu et al., (2024) found that high Ezrin expression was associated with a greater number of metastatic foci in a nude mouse liver metastasis model. Li T. et al., (2019) further demonstrated that MST4 promoted *in vivo* metastasis of gastric cancer by interacting with Ezrin and enhancing Ezrin phosphorylation. In more recent studies, Mesquita et al., (2025) reported that depletion of YTHDF3 impaired the motility of gastric cancer cells and reduced the formation of lung metastases, with Ezrin identified as a relevant downstream target.

In this study, we first analyzed the differential expression of *SLC12A7* in human GC tissue and adjacent tissues using TCGA and GEO databases, established a prognostic model, and analyzed its clinical and pathological correlation. Then, following confirmation of the significant upregulation of SLC12A7 in GC cells, we knocked down SLC12A7 and reduced its expression levels in MGC803 and HGC27 GC cells, exploring the impacts of altered SLC12A7 expression on biological behaviors such as proliferation, migration, invasion, apoptosis, and cell cycle progression. Meanwhile, we investigated the influence of the *SLC12A7* knockdown on the expression levels of EMT marker proteins including E-Cadherin and Vimentin. Finally, we explored whether Ezrin-associated PI3K/AKT signaling participates in the malignant phenotypes regulated by *SLC12A7* in GC cells.

## Methods and materials

### Data collection

mRNA expression profiles and clinical pathological characteristic data were obtained from TCGA database (https://tcga-data.nci.nih.gov/tcga/), including data from 415 gastric cancer samples and 35 adjacent non-tumor samples, exclude samples from different batches of the same patient, and download the clinical data. Datasets GSE29272 (n = 268), GSE3438 (n = 99), GSE66229 (n = 400) were obtained from GEO database (https://www.ncbi.nlm.nih.gov/geo/), further validating the differentially expression of target gene.

### Survival analysis and clinical analysis

Using data obtained from TCGA database and GEO database, all data were divided into *SLC12A7* high expression group and *SLC12A7* low expression group based on the optimal cutoff value as a basis for constructing a prognostic model of *SLC12A7*. With a total of 450 samples included in the analysis, the categorical variables were compared between the *SLC12A7* high expression group and *SLC12A7* low expression group using chi square test, numerical variables were compared using t-test. P < 0.05 was considered statistically significant.

### Gene interaction analysis

Ezrin molecules interacting with *SLC12A7* were identified via the STRING database. Gene correlation analysis was performed using 450 data from the TCGA and 767 data from the GEO databases. Docking experiments between *SLC12A7* and Ezrin proteins were conducted on the HADDOCK 2.4 web server. The RMSD was stabilized at < 2.1 Å, with >85% retention of interfacial hydrogen bonds and salt bridges, confirming thermodynamic validity of the docked conformation.

### Tissue samples collection

Tissue microarrays containing 160 paraffin-embedded tissue cores from 80 patients, including paired tumor and adjacent non-tumor tissues, were used for immunohistochemical analysis. In addition, 47 pairs of fresh-frozen GC and adjacent non-tumor tissues were collected from patients who underwent surgical treatment at Shengjing Hospital of China Medical University. This study has been approved by the Ethics Committee of Shengjing Hospital of China Medical University (2023PS422 K(T2)). All patient samples meet the criteria of pathological diagnosis, and the preoperative and postoperative diagnosis results are clear as primary GC. The samples meet the standard of performing GC radical gastrectomy, including not receiving special treatments before surgery, such as chemotherapy, radiation therapy, molecular targeted therapy, as well as immunotherapy.

### Cell lines and reagents

Gastric cancer cell lines MGC803, BGC823, HGC27 and normal gastric epithelial cell line GES-1 were purchased from Nanjing KeyGEN biotech Co., Ltd. Cells were cultured in RPMI-1640 medium (E600028, Sangon Biotech, Shanghai, China) containing 10% fetal bovine serum (E600001, Sangon Biotech) and 1% penicillin-streptomycin solution (RC17032, Hyclone). All cells were stored in an incubator at 37 °C and 5% CO_2_.

### Quantitative real-time PCR

Total mRNA of different cells was extracted using RNAiso Plus reagent (D9108B, Takara, Beijing, China) according to the manufacturer’s protocol. Synthesis of cDNA was completed using M-MuLV First Strand cDNA Synthesis Kit (B532435, Sangon Biotech) according to the manufacturer’s protocol. The process of qRT-PCR utilized 2×SG Fast qPCR Master Mix (B639271, Sangon Biotech), forward and reverse primers are listed in Supplementary Material S1, template cDNA, and PCR-grade water. Data were calculated using the 2^−ΔΔCT^ method.

### Immunohistochemistry

Immunohistochemistry (IHC) was performed using small sections of tissues. All tissue samples were deparaffinized in xylene and rehydrated utilizing descending concentrations of ethanol. The slides were processed by microwave heating for antigen retrieval. The slides were then processed using 3% hydrogen peroxide to prevent endogenous peroxidase. After washed twice using PBS, the slides were incubated overnight with anti-*SLC12A7* (26590-1-AP, 1:250, Proteintech) at 4 °C. Corresponding secondary antibody was applied at room temperature and treated for 30 min. Further, the tissue sections were stained with 3, 3-diaminobenzidine (DAB), after washed for 5 min, slides were stained with hematoxylin for 1 min at room temperature.

### Transfection of small interfering RNA

To downregulate the expression of *SLC12A7*, siRNA against *SLC12A7* (si-*SLC12A7*, GenePharma, Shanghai, China) was transfected into HGC27 and MGC803 cells (Supplementary Material S2). GP-transfect-Mate (GenePharma) was applied according to the manufacturer’s instructions during the transfection of si-*SLC12A7* and siRNA of negative control (si-NC, GenePharma). After 48 h and 72 h of transfection, mRNA and protein were collected to detect transfection efficiency via qRT-PCR and Western blot, all assays were repeated in triplicate.

### Cell viability assay

CCK-8 kit (K1018, APExBio, United States) was used for the cell viability test. A total of 2 × 10^3^ HGC27 and MGC803 cells were placed in each well of a 96-well plate after knockdown using si-*SLC12A7* or si-NC. At 24 h, 48 h, 72 h, and 96h, 10 µL of the CCK-8 reagent was added into each well and incubated at 37 °C. A microplate reader (Spark 10M, Tecan, Männedorf, Switzerland) was used to measure the absorbance at a 450 nm wavelength at 1h, 2h, and 3 h.

### Transwell migration and invasion assay

Transwell chambers (#3422, Costar, Corning, NY, United States) were used to detect the migration and invasion capabilities. For Transwell migration assay, a total of 1 × 10^5^ HGC27 and MGC803 cells transfected with si-*SLC12A7* and si-NC were seeded in Transwell chambers with 200 µL serum-free RPMI-1640 medium. For Transwell invasion assay, matrigel (#356234, Becton, Dickinson and Company, United States) was diluted using serum-free medium (1:8). A total of 2 × 10^5^ HGC27 and MGC803 cells transfected with si-*SLC12A7* and si-NC were seeded in Transwell chambers with 60 µL diluted matrigel and incubated at 37 °C. Another 600 µL RPMI-1640 medium with 10%FBS was added as a chemoattractant in corresponding 24-well plate.

After incubation for 48 h, chambers were washed using PBS and cells were fixated using 4% paraformaldehyde for 15 min at room temperature. Chambers were dyed with 0.25% crystal violet solution for 20 min after cleaning paraformaldehyde. Photomicrographs of the Transwell chambers were taken using an inverted microscope (ECLIPSE Ts2, Nikon, Tokyo, Japan) and the number of migrated cells was counted using ImageJ software.

### Flow cytometry

For cell apoptosis rate detection, HGC27 and MGC803 cells were pretreated separately using si-*SLC12A7* or si-NC for 48 h. A total of 1 × 10^6^ cells at the logarithmic phase of growth were harvested according to manufacturer’s protocol: rinsed twice with PBS, each sample resuspended using a 100 µL 1×binding buffer, 5 µL of FITC-Annexin V and 5 µL of propidium iodide (PI) (MK1028, Boster, Wuhan, China) added and incubated on ice in the dark for 15 min. Another 400 µL of 1×binding buffer was added to resuspend the cells. Apoptosis rates were detected in a FACS Celesta flow cytometer (Becton Dickinson and Company, Franklin Lakes, NJ, United States); 1 × 10^4^ events per sample were analyzed using MODFIT software (Becton Dickinson and Company).

For cell cycle detection, a total of 1 × 10^6^ cells at the logarithmic phase of growth were harvested, then rinsed twice with PBS, and fixed with 70% cold ethanol. The cells were then stained with 0.5 mL PI (C1052, Beyotime, Shanghai, China) in dark for 30 min. A FACS Calibur flow cytometer (Becton Dickinson and Company) was employed to detect the cell cycle, which was then analyzed using MODFIT software (Becton Dickinson and Company).

### Western blot

All proteins were collected at 4 °C. BCA assay was performed previously to quantify protein concentration. A total of 20 μg of proteins was isolated via SDS-PAGE and transferred to 0.45 µm polyvinylidene fluoride (PVDF) membranes (BS-PVDF-45, Biosharp, Hefei, China). Then, the membranes were blocked with 5% non-fat milk for 1 h at room temperature and incubated with primary antibodies at 4 °C overnight, followed by incubation with HRP secondary antibodies for 1 h at room temperature. Primary antibodies included anti-*SLC12A7* (1:2000; 26590-1-AP Proteintech, Wuhan, China), anti-Vimentin (1:1,000; PB9359, Boster), anti-E-Cadherin (1:1,000; PB9561, Boster), anti-Caspase-9 (1:1,000, BM4619, Boster), anti-Caspase 3 (1:1,000, BM3957, Boster), anti-CDK1 (1:500, M00209-2, Boster), anti-Cyclin A2 (1:500, PB9424, Boster), anti-PI3K (1:1,000, ab302958, Abcam, United States), anti-p-PI3K (p85α, pY607; 1:1,000, ab182651, Abcam), anti-AKT (1:500, sc-5298, Santa Cruz Biotechnology, United States), anti-p-AKT (1:500, sc-293125, Santa Cruz Biotechnology), anti-Ezrin (1:5,000, ab40839, Abcam) and anti-GAPDH (1:5,000; 60004-1-Ig, Proteintech) was utilized for normalization.

### Immunofluorescence co-localization

To examine the expression and localization of *SLC12A7* and Ezrin in gastric cancer cells multiplex immunofluorescence staining was performed using a two-color TSA-based mIHC kit (IHCT001, Bioss Antibodies, China) according to the manufacturer’s instructions. MGC803 and HGC27 cells were seeded onto coverslips placed in 6-well plates and cultured at 37 °C, 5% CO_2_ for 24 h. The cells were then fixed with 4% paraformaldehyde at room temperature for 20 min, permeabilized with 0.3% Triton X-100 for 20 min, followed by incubation in a 3% hydrogen peroxide solution at room temperature in the dark for 15 min, and blocked with 3% bovine serum albumin (BSA) for 30 min to prevent non-specific binding. Subsequently, the cells were incubated at room temperature with the primary antibody against *SLC12A7* (1:200, 26590-1-AP, Proteintech, China) for 1 hour. After three washes with PBS, the HRP-conjugated goat anti-rabbit/mouse universal secondary antibody provided in the kit was applied and incubated at room temperature for 1 h. Following secondary antibody incubation, the slides were stained with TSA-520PLus fluorescent dye for 15 min. After staining, the slides were incubated with antibody elution buffer (P1453, Beyotime Biotechnology, China) at 37 °C for 10 min. The elution buffer was removed, fresh elution buffer was added, and the slides were incubated again at 37 °C for another 10 min to remove the bound antibody complex. After antibody elution, the slides were incubated with an anti-Ezrin primary antibody (1:200, 26056-1-Ap, Proteintech, China) under the same conditions as described above. After incubation with the HRP-conjugated secondary antibody, the slides were stained with TSA-570PLus fluorescent dye for 15 min. After completion of the co-staining procedure, nuclei were counterstained with ready-to-use DAPI stain in the dark at room temperature for 5 min. Finally, the slides were mounted with an anti-fade mounting medium. Fluorescent signals were observed using a confocal microscope (FV3000, Olympus, Japan), and the Pearson correlation coefficient for the colocalization of *SLC12A7* and Ezrin was analyzed using ImageJ.

### Statistics analysis

Chi-squared tests were used to assess associations between SLC12A7 expression and clinicopathological characteristics of gastric cancer. SLC12A7 high expression was defined by the median expression level in TCGA data and by the 47th percentile (determined by x-tile) of the tumour-to-adjacent tissue immunohistochemical score ratio in tissue microarray data. Other experimental data were analyzed using GraphPad Prism 8.0 software (GraphPad Software Inc., La Jolla, CA, United States). Two sets of data were analyzed using t-test, and two or more sets of data were analyzed using one-way ANOVA. All data were expressed as mean ± SEM, with P < 0.05 indicating statistical differences (ns represents not significant; * represents p < 0.05; ** represents p < 0.01; *** represents p < 0.001; **** represents p < 0.0001).

## Results

### *SLC12A7* is highly expressed in gastric cancer tissues and is positively correlated with poor patient prognosis

The solute carrier protein family was found to be closely related to the occurrence and development of human cancer, suggesting that *SLC12A7* may be one of the key oncogenes in GC. Pan-cancer expression analysis of *SLC12A7* was performed using the TIMER database, and the results are shown in Figure 1A. *SLC12A7* was significantly overexpressed in breast invasive carcinoma, cholangiocarcinoma, esophageal carcinoma, head and neck squamous cell carcinoma, kidney renal clear cell carcinoma, hepatocellular carcinoma, lung adenocarcinoma, prostate adenocarcinoma, stomach adenocarcinoma, and uterine corpus endometrial carcinoma, with stomach adenocarcinoma being particularly significant.

**FIGURE 1:**
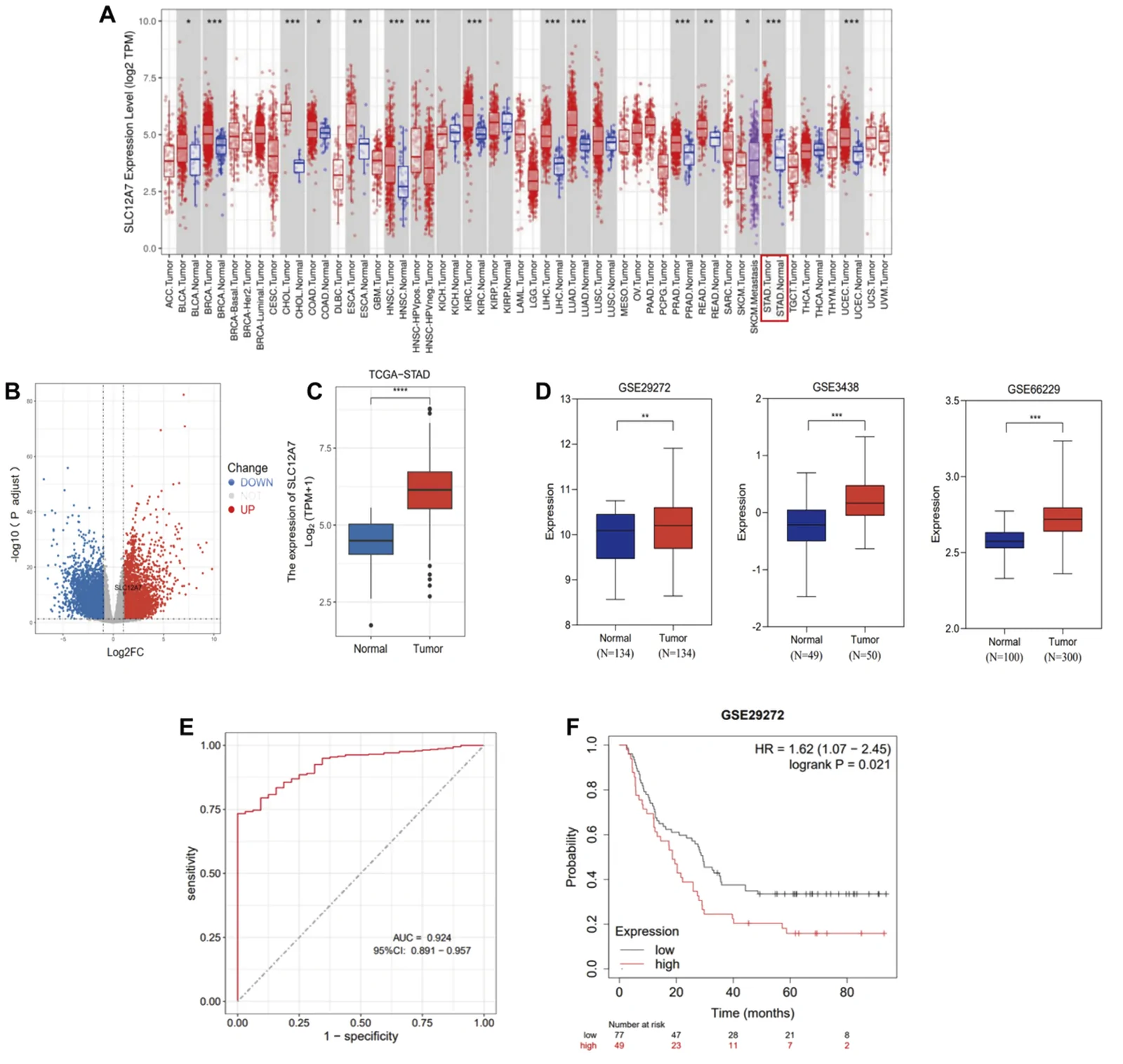
Bioinformatic analysis of SLC12A7 **(A)** Pan-cancer analysis results of *SLC12A7* overexpression in multiple types of cancer **(B,C)** TCGA database indicated *SLC12A7* overexpression in GC tissue samples and adjacent tissue samples **(D)** SLC12A7 mRNA expression was higher in GC tissues than in non-tumor tissues in GSE29272, GSE3438, GSE66229 datasets **(E,F)** ROC curve and OS analysis of *SLC12A7*, indicating high *SLC12A7* expression correlated with poor prognosis. ** represents p<0.01, *** represents p<0.001, ****represents p<0.0001.

Clinical data of 450 GC patients were obtained from the TCGA database, then analyzed and plotted a volcano plot. Figure 1B show that the expression level of *SLC12A7* was significantly upregulated (|log2FC|>1, *p* < 0.05). According to TCGA-STAD transcriptome data, when compared with adjacent cancer tissues, the mRNA expression of *SLC12A7* is significantly upregulated in GC tissues (Figure 1C, *p* < 0.0001). In order to further validate the mRNA expression level of *SLC12A7*, 3 GC datasets, GSE29272, GSE3438, and GSE66229, were obtained from the GEO database for analysis. The results were consistent with the analysis results of the TCGA database, which means the transcription level of *SLC12A7* in GC tissue was significantly increased (Figure 1D).

We next evaluated the prognostic value of SLC12A7 expression in GC. AUC of ROC curve in TCGA was 0.924 (Figure 1E). The analysis of the GSE29272 dataset proved that the overall survival rate of patients in the high *SLC12A7* expression group is significantly lower than that of patients in the low *SLC12A7* expression group (Figure 1F, *p* < 0.05). The results suggested that SLC12A7 overexpression might correlate with a worse prognosis in GC.

### Correlation between *SLC12A7* expression and clinical pathological factors

To further investigate the clinical pathological factors leading to poor prognosis in GC patients, clinical data from 450 GC patients were obtained from the TCGA database and extracted relevant data including age, gender, histological grading, staging, TNM staging, and survival time to analyze the relationship between *SLC12A7* expression levels and clinical pathological factors. The results suggested that the expression level of *SLC12A7* was significantly correlated with distant metastasis (p = 0.027) and differentiation degree (p = 0.001) in GC patients (Table 1).

**TABLE 1:** Correlation between SLC12A7 mRNA expression and clinical pathological factors of GC patients in TCGA database.

Clinical pathological factors	Cases (*n*)	SLC12A7 high (n,%)	SLC12A7 low (n,%)	*P-value*
Age	​	​	​	0.09
≤67	231	106 (23.6)	125 (27.8)	
>67	219	119 (26.4)	100 (22.2)	
Gender	​	​	​	0.076
Female	159	70 (15.6)	89 (19.8)	
Male	291	155 (34.4)	136 (30.2)	
Tumor invasion	​	​	​	0.296
T1	24	14 (3.2)	10 (2.2)	
T2	100	49 (11.1)	51 (11.6)	
T3	197	105 (23.8)	92 (20.9)	
T4	120	52 (11.8)	68 (15.4)	
Lymph node status	​	​	​	0.761
N0	135	67 (15.6)	68 (15.8)	
N1	125	59 (13.7)	66 (15.3)	
N2	88	48 (11.2)	40 (9.3)	
N3	82	42 (9.8)	40 (9.3)	
Distant metastasis	​	​	​	0.027*
M0	398	202 (47.3)	196 (45.9)	
M1	29	8 (1.9)	21 (4.9)	
TNM stages	​	​	​	0.099
I	62	34 (8.0)	28 (6.6)	
II	141	63 (14.9)	78 (18.4)	
III	176	96 (22.6)	80 (18.9)	
IV	45	17 (4.0)	28 (6.6)	
Grade	​	​	​	0.001*
G1	12	5 (1.1)	7 (1.6)	
G2	162	100 (22.7)	62 (14.1)	
G3	267	116 (26.3)	151 (34.2)	

Bold type and * indicate statistically significant (p < 0.05).

The tissue microarrays applied in this research included 80 pairs of paraffin samples from GC and corresponding adjacent tissues of 80 patients. Clinical and pathological data of these patients were collected and analyzed. After excluding 3 cases with incomplete tissue specimens, the analysis included a total of 77 patient samples. SLC12A7 protein expression in human GC tissues was evaluated by immunohistochemistry. The results showed that expression of *SLC12A7* in GC tissues was significantly higher than adjacent tissues (Figures 2A,B).

**FIGURE 2:**
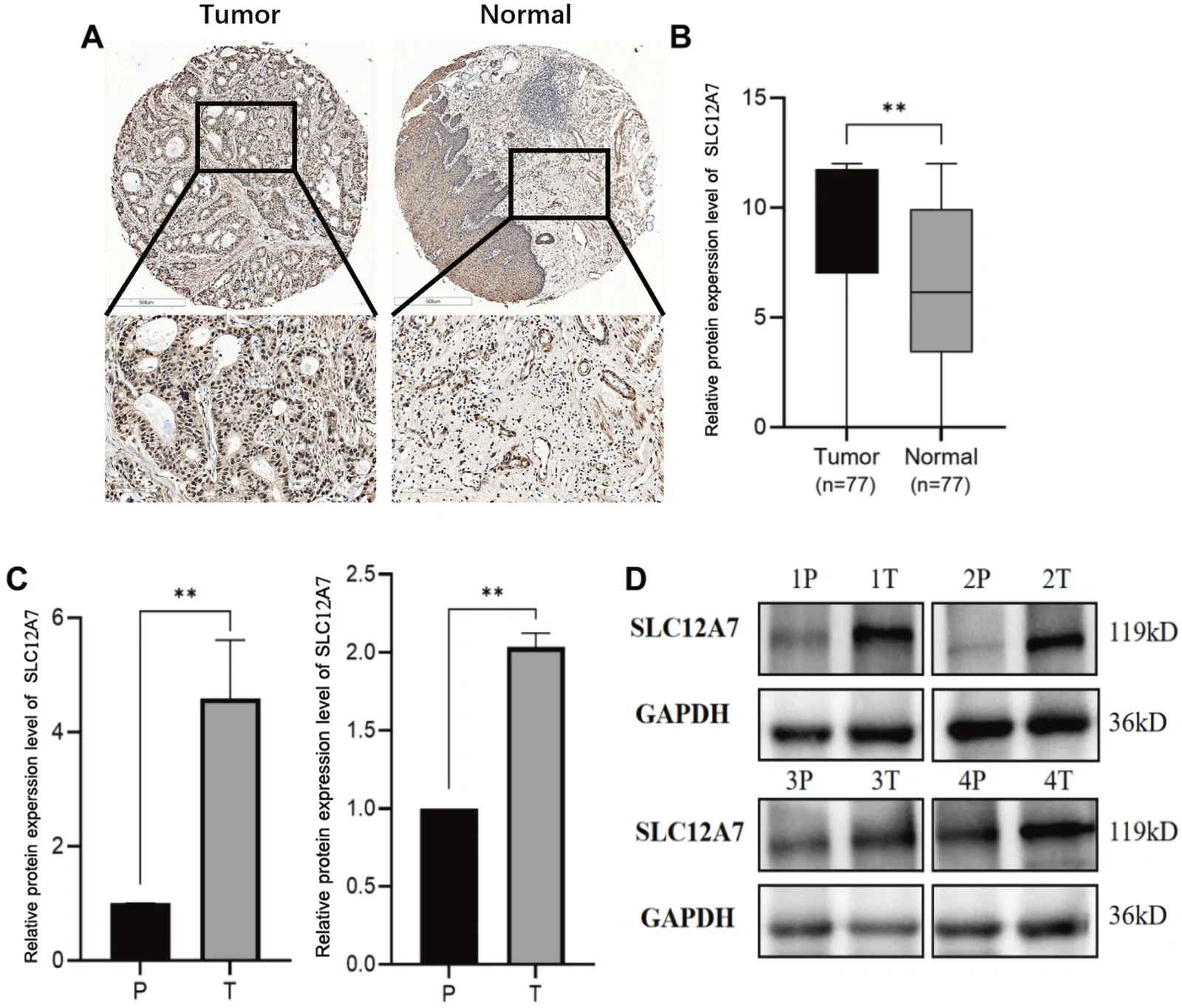
Overexpression of *SLC12A7* in gastric cancer tissue samples compared to adjacent tissue samples **(A)** Immunohistochemistry detection of *SLC12A7* staining for GC and adjacent tissues in GC tissue microarrays **(B)** *SLC12A7* expression score chart in 77 pairs of GC and adjacent tissue samples **(C)** qRT-PCR indicated of mRNA expression level of *SLC12A7* in GC tissues and corresponding adjacent tissues **(D)** Western blot was conducted on 47 pairs of GC and corresponding adjacent tissues to investigate the expression of *SLC12A7* protein in GC tissues and adjacent tissues. All assays were performed in triplicate (** represents *p* < 0.01, P stands for adjacent tissues, T stands for GC tissues).

After analyzing clinical medical records of 77 pairs of gastric cancer patients and the relationship between *SLC12A7* expression and clinical pathological factors, we discovered that SLC12A7 expression was significantly associated with gender (P = 0.025) and tumor invasion status (P = 0.037), whereas no significant associations were observed with age, lymph node status, distant metastasis, TNM stage, tumor size, tumor location, or survival status (Table 2).

**TABLE 2:** Correlation between expression level of *SLC12A7* and clinical pathological factors of GC patients in tissue microarrays.

Clinical pathological factors	Cases(n)	SLC12A7 high	SLC12A7 low	P-value
Gender				0.025*
Male	51	22	29	
Female	26	19	7	
Age				0.411
≤58	37	22	15	
>58	40	19	21	
Tumor invasion (T)				0.037*
T1	8	6	2	
T2	17	4	13	
T3	2	1	1	
T4	50	30	20	
Lymph node (N)				0.607
N0	27	15	12	
N1	17	9	8	
N2	21	9	12	
N3	12	8	4	
Distant metastasis (M)				1
M0	72	38	34	
M1	5	3	2	
TNM Stage				0.69
I	17	7	10	
II	20	12	8	
III	35	19	16	
IV	5	3	2	
Tumor size				0.998
≤4 cm	46	25	21	
>4 cm	31	16	15	
Pathogenic site				0.361
Gastric antrum	38	23	15	
Lesser curvature	19	11	8	
Gastric body	8	3	5	
Cardia	8	2	6	
Unknown	4	2	2	
Survival status				1
Alive	35	19	16	
Dead	42	22	20	

*p < 0.05.

We also performed qRT-PCR and Western blot on 47 pairs of frozen GC and adjacent non-tumor tissue samples collected from Shengjing Hospital of China Medical University. The results indicated that mRNA and protein expression levels of *SLC12A7* were significantly elevated in GC tissue samples compared with adjacent tissue samples (Figures 2C,D).

### Knockdown of *SLC12A7* inhibited proliferation, migration, and EMT process in GC cell lines

This research selected 2 human GC cell lines HGC27, MGC803 and 1 normal stomach epithelial cell line GES-1. *SLC12A7* expression levels were measured in the cell lines by qRT-PCR and Western blot. Quantify and standardize grayscale values using ImageJ software. mRNA and protein expression levels of *SLC12A7* were prominently higher in GC cell lines than that in normal stomach epithelial cell line (Figures 3A–C).

**FIGURE 3:**
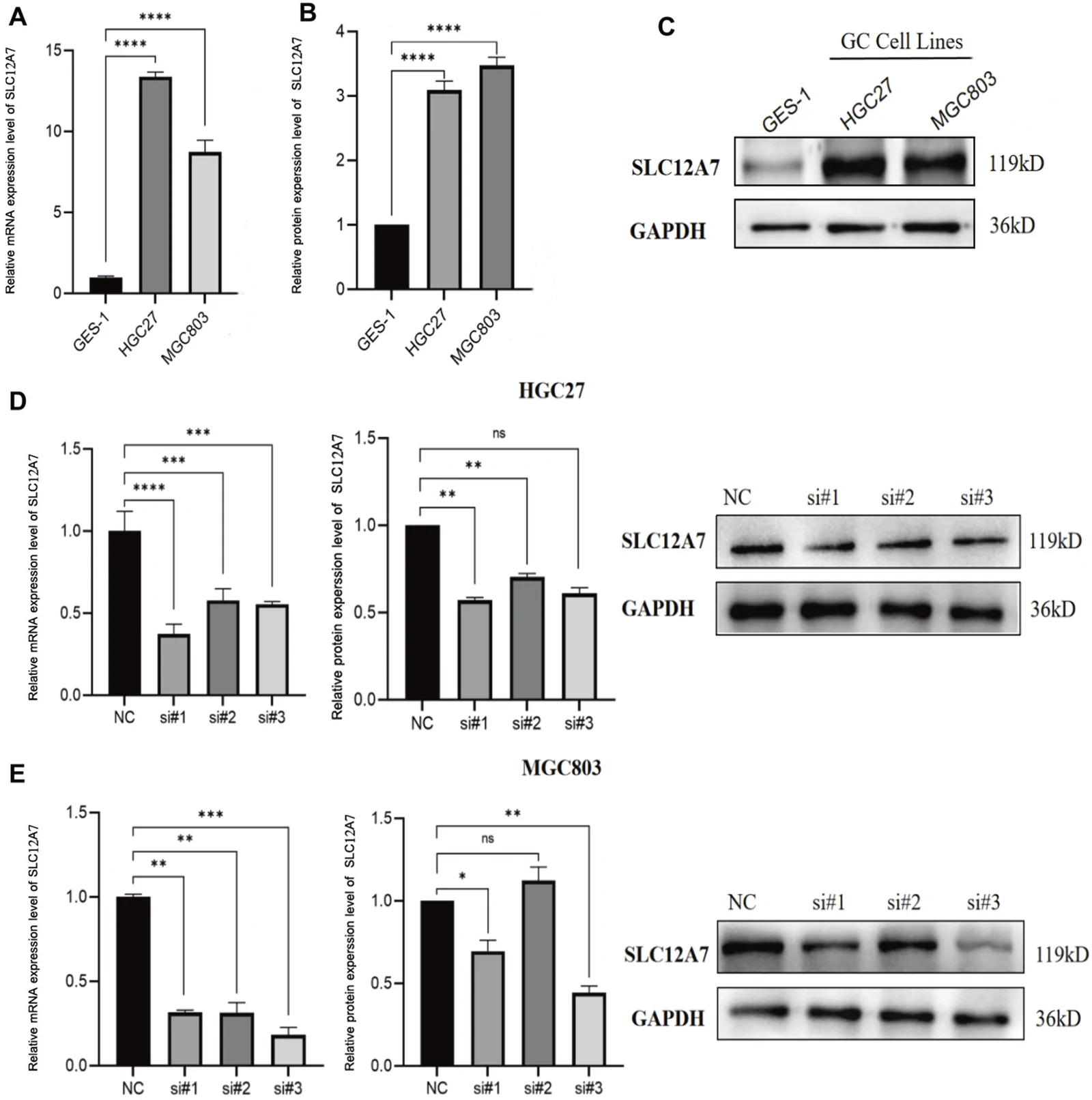
Expression of *SLC12A7* in gastric cancer cell lines before and after siRNA knockdown **(A–C)** mRNA expression levels and protein expression levels of *SLC12A7* were elevated in all 2 GC cell lines HGC27, MGC803 compared with normal stomach epithelial cell line GES-1 **(D)** mRNA expression levels and protein expression levels of *SLC12A7* in HGC27 cells **(E)** mRNA expression levels and protein expression levels of *SLC12A7* in MGC803 cells. All assays were performed in triplicate (ns represents not significant; * represents p < 0.05; ** represents p < 0.01; *** represents p < 0.001; **** represents p < 0.0001).

After siRNA transfection, we applied qRT-PCR and Western blot to verify the efficacy. The result showed that in HGC27 cell line si#1 was more effective, and that in MGC803 cell line si#3 was more effective. Therefore, we selected si#1 and si#3 to perform the subsequent assays (Figures 3D,E).

CCK-8 assay was performed to measure the impact of *SLC12A7* knockdown in HGC27 and MGC803 cells. The results presented a significant inhibition of proliferation in HGC27 and MGC803 cell lines (Figures 4A,B).

**FIGURE 4:**
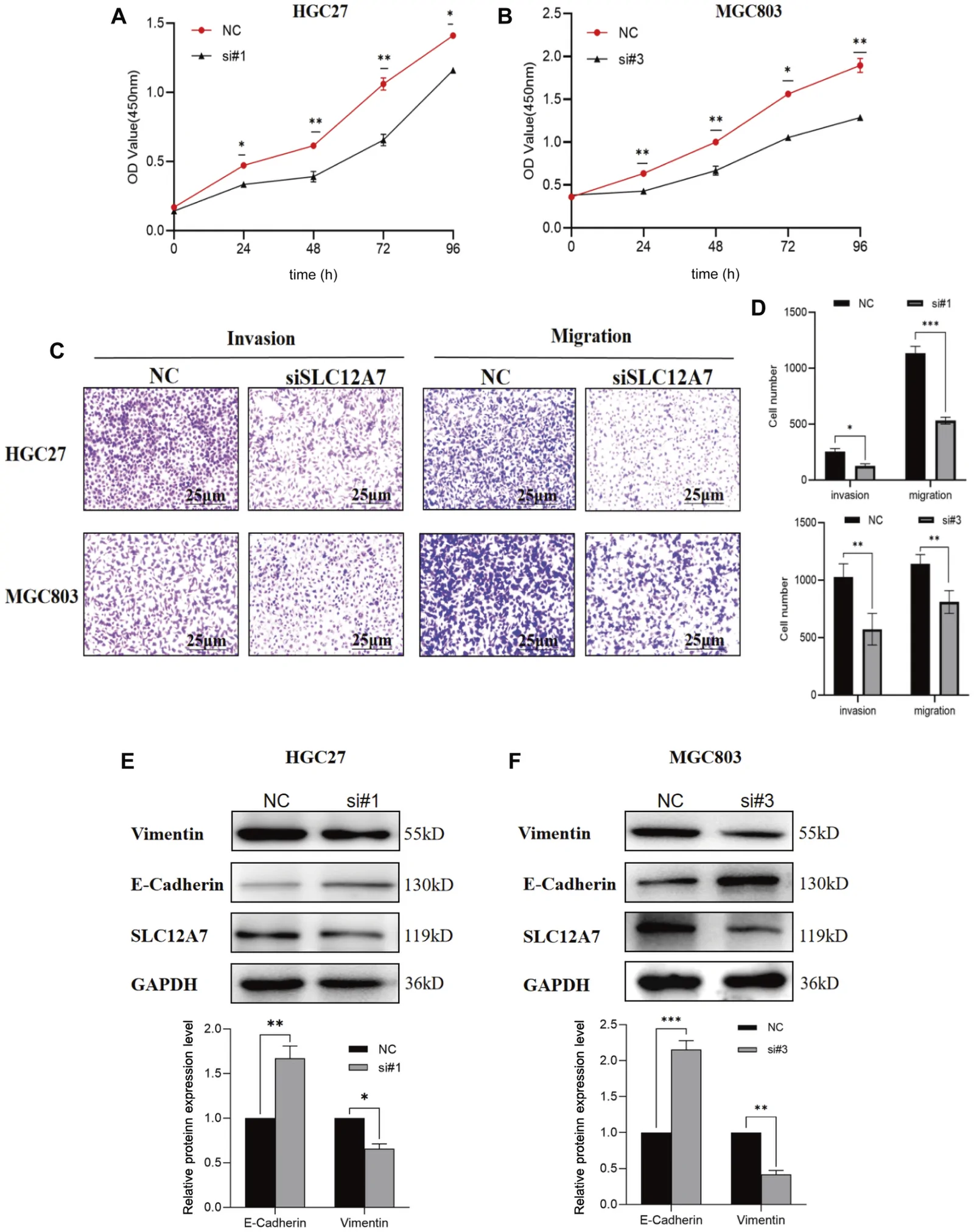
Knockdown of *SLC12A7* inhibited proliferation, metastasis, and EMT process in GC cell lines **(A,B)** Knockdown of *SLC12A7* led to cancer proliferation inhibition in HGC27 and MGC803 cell lines **(C,D)** Knockdown of SLC12A7 reduced the migration and invasion of GC cells. **(E,F)** Knockdown of *SLC12A7* exhibited prominent E-Cadherin upregulation and Vimentin downregulation. All assays were performed in triplicate (* represents *p* < 0.05; ** represents *p* < 0.01; *** represents *p* < 0.001).

Cancer cell metastasis is closely related to its capabilities of migration and invasion, this biological process is widely regarded as a common cause of cancer-associated death. Transwell migration and invasion assays revealed that knockdown of SLC12A7 significantly inhibited the migration and invasion of HGC27 and MGC803 cells (Figures 4C,D).

EMT enables cancer cells to acquire mesenchymal features and enhances migratory and invasive potential. After *SLC12A7* knockdown, we measured the expression of EMT target protein E-Cadherin and Vimentin via Western blot. Compared with control group, *SLC12A7* knockdown group exhibited E-Cadherin upregulation and Vimentin downregulation (Figures 4E,F), suggesting *SLC12A7* may participate in the modulation of EMT process in GC cells.

### Knockdown of *SLC12A7* arrested GC cell cycle and accelerated GC apoptosis

To better understand the role of SLC12A7 in cell-cycle regulation, the expression of the cell-cycle-regulating proteins CDK1 and Cyclin A2 was measured by Western blot. Compared to negative control group, CDK1 and Cyclin A2 were both downregulated in HGC27 and MGC803 (Figures 5A,B).

**FIGURE 5:**
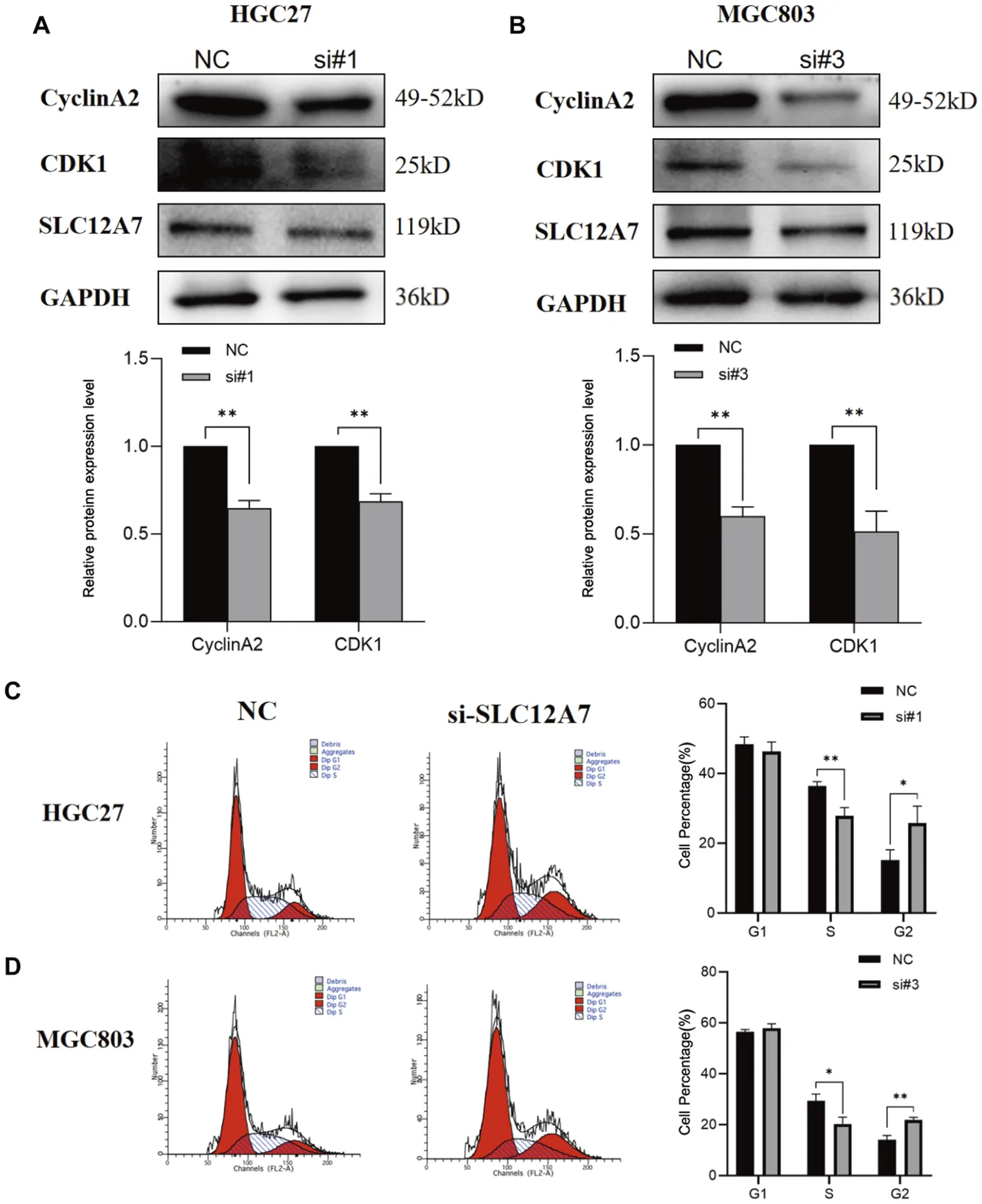
Knockdown of *SLC12A7* led to cell cycle arrest **(A,B)** CDK1 and Cyclin A2 were downregulated in HGC27 and MGC803 cells after *SLC12A7* inhibition **(C,D)** Flow cytometry results indicated an increase in the percentage of G2-phase cells and a decrease in the percentage of S-phase cells after SLC12A7 knockdown, suggesting G2/M cell-cycle arrest. All assays were performed in triplicate (* represents *p* < 0.05; ** represents *p* < 0.01).

Flow cytometry was then applied to investigate cell cycle transition. Compared to negative control group, *SLC12A7* knockdown in HGC27 cells significantly reduced the percentage of S phase cells from (36.41 ± 1.03)% to (27.89 ± 1.92)%, while the percentage of G2 phase cells increased from (15.16 ± 2.40)% to (25.78 ± 3.95)% (Figure 5C), in MGC803 cell line percentage of S phase cells reduced from (29.45 ± 2.11)% to (20.30 ± 2.19)%, while percentage of G2 phase cells increased from (14.07 ± 1.04)% to (21.8 ± 0.87)% (Figure 5D). The results suggested that knockdown of *SLC12A7* may induce G2/M cell-cycle arrest, thus inhibiting GC proliferation.

Flow cytometry analysis was conducted to indicate the apoptosis rates after *SLC12A7* knockdown. It was observed that the apoptosis rates in *SLC12A7* knockdown HGC27 cells and MGC803 cells were significantly higher than those in the control group. In HGC27 cells the apoptosis rates increased from (19.97 ± 0.71)% to (27.27 ± 4.01)%, while in MGC803 cells the apoptosis rates increased from (9.69 ± 0.58)% to (16.63 ± 2.05)% (Figure 6A).

**FIGURE 6:**
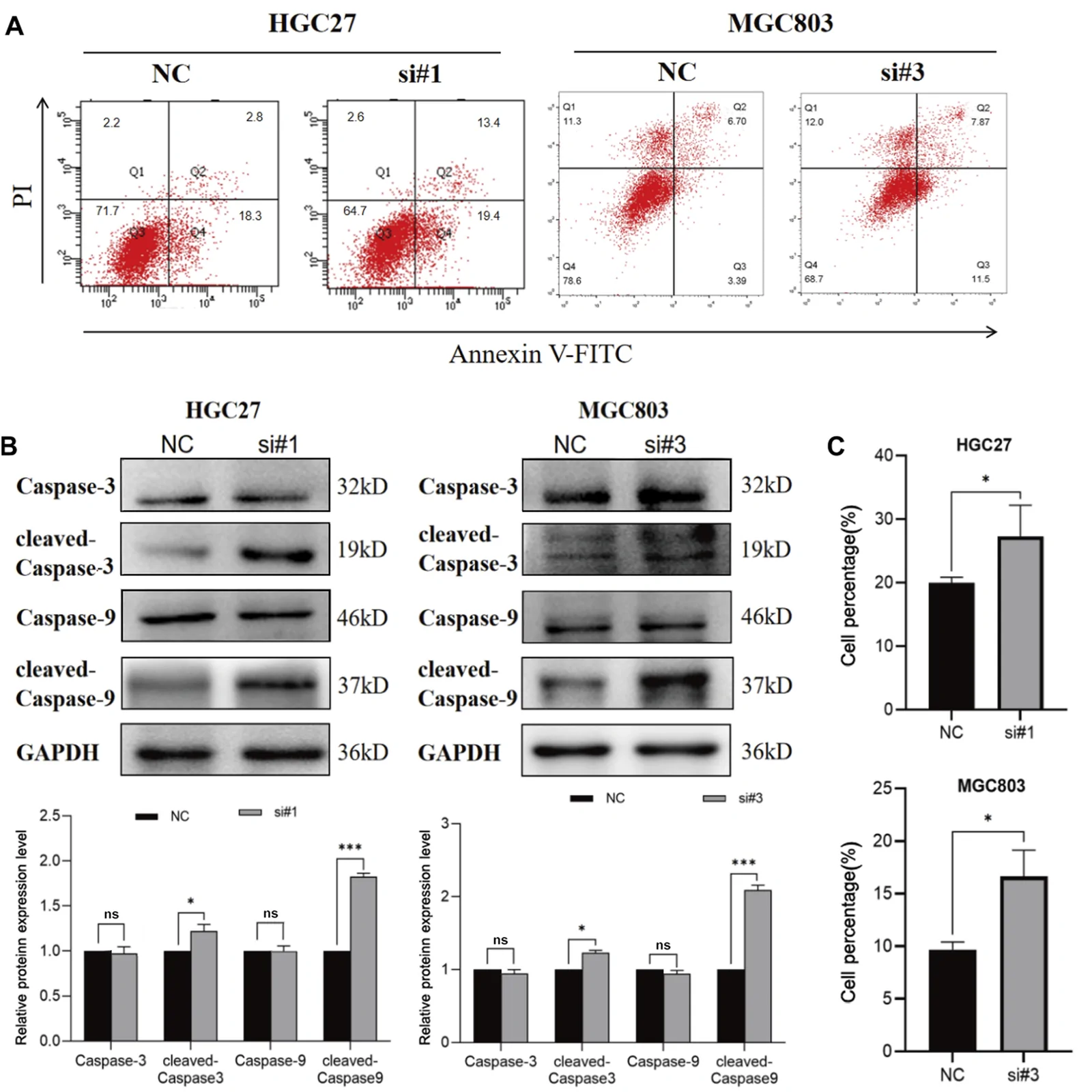
Knockdown of *SLC12A7* resulted in apoptosis in GC cells **(A,C)** Flow cytometry results showed elevated apoptosis rates in HGC27 and MGC803 cells after application of siRNA inhibition **(B)** Expression levels of apoptosis-related protein in HGC27 and MGC803 cells after *SLC12A7* inhibition. All assays were performed in triplicate (ns represents not significant; * represents *p* < 0.05; *** represents *p* < 0.001; **** represents *p* < 0.0001).

Western blot results of apoptosis-associated proteins showed that the expression of cleaved Caspase 9 and cleaved Caspase 3 increased after *SLC12A7* knockdown in both cell lines, which further validated the results of flow cytometry analysis (Figures 6B,C). Overall, the inhibition of *SLC12A7* expression led to a decrease in GC viability.

### *SLC12A7* knockdown attenuates PI3K/AKT phosphorylation and Ezrin expression in gastric cancer cells

Western blot was performed to investigate the effect of *SLC12A7* knockdown on the expression of PI3K/AKT signaling pathway related proteins. Compared to negative control group, *SLC12A7* knockdown in HGC27 cells and MGC803 cells resulted in lower p-PI3K and p-AKT levels (*p* < 0.05), whereas PI3K and AKT levels showed no significant changes (Figures 7A,B).

**FIGURE 7:**
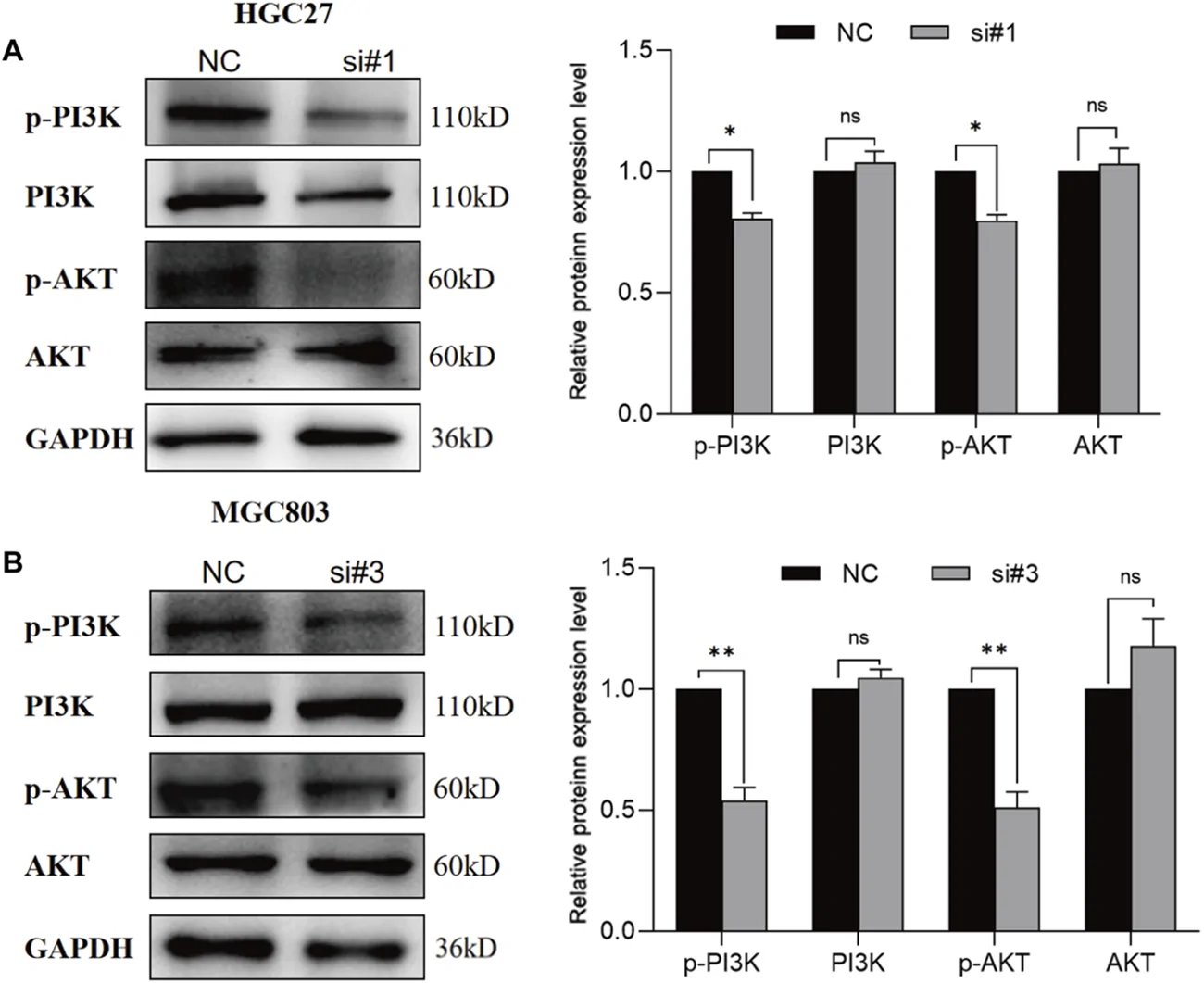
Knockdown of *SLC12A7* influenced PI3K/AKT signaling pathway in GC cells **(A,B)** Western blot results of PI3K and AKT total protein expression levels and phosphorylated protein expression levels in HGC27 and MGC803 cells after knocking down *SLC12A7*. All assays were performed in triplicate (ns represents not significant; * represents *p* < 0.05; ** represents *p* < 0.01).

The correlation analysis between *SLC12A7* and Ezrin expression levels showed a significant positive correlation (Figure 8A, r = 0.3, *p* < 0.0001). Molecular docking method was applied to simulate the interaction mode between *SLC12A7* and Ezrin, with a predicted binding energy of –86.7038 kcal/mol (Figure 8E). Molecular docking predicted a potentially favorable binding conformation between *SLC12A7* and Ezrin. Consistently, immunofluorescence costaining of *SLC12A7* and Ezrin in gastric cancer cell lines MGC803 and HGC27 showed partial colocalization (Figure 8F). Apart from lower p-PI3K and p-AKT expression levels, knockdown of *SLC12A7* also reduced Ezrin level (Figure 8B, *p* < 0.05). Ezrin may be involved in *SLC12A7*-associated signaling regulation. Co-transfection of Ezrin overexpression plasmid and si-*SLC12A7* in HGC27 and MGC803 cells partly restored the phosphorylation levels of PI3K and AKT, as well as Ezrin protein levels in HGC27 and MGC803 cells, while there was no significant change in the total protein levels of PI3K and AKT (Figures 8C,D). These results demonstrate that *SLC12A7* is positively correlated with Ezrin at both the expression and subcellular levels, and that the two proteins may functionally cooperate in PI3K-AKT signaling activation.

**FIGURE 8:**
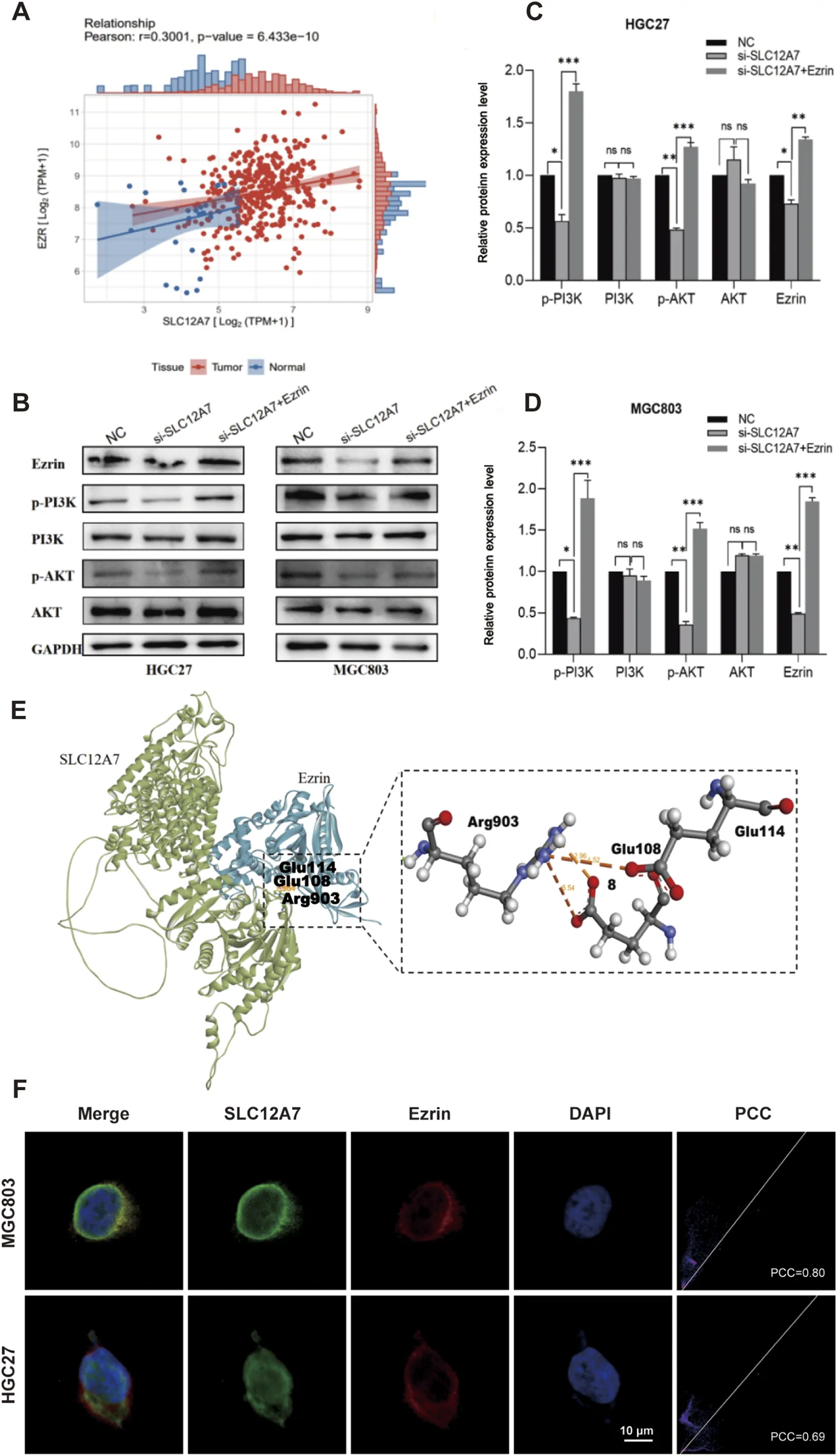
*SLC12A7* knockdown attenuates PI3K/AKT phosphorylation and Ezrin expression in gastric cancer cells **(A)** Correlation analysis between *SLC12A7* and Ezrin **(B–D)** Western blot analysis of PI3K, p-PI3K, AKT, p-AKT, and Ezrin protein expression levels in HGC27 and MGC803 cells after co-transfected with si-*SLC12A7* and Ezrin **(E)** Docking simulation of *SLC12A7* and Ezrin molecules. All assays were performed in triplicate (ns represents not significant; * represents *p* < 0.05; ** represents *p* < 0.01; *** represents *p* < 0.001) **(F)** *SLC12A7* and Ezrin show immunofluorescent colocalization in MGC803 (PCC = 0.80) and HGC27 (PCC = 0.69).

## Discussion

Gastric cancer is the fifth most common cancer worldwide and ranks third among all malignant tumors in terms of mortality (Sung et al., 2021). Gastric cancer has a high degree of heterogeneity at the molecular level. Exploring the important molecules which regulate the progression of gastric cancer will provide valuable targets for cancer diagnosis and treatment. Therefore, it is crucial to find potential tumor markers which contribute to clinical treatment of gastric cancer.

*SLC12A7* is a 1083-amino acid transmembrane protein which regulates cell volume through transporting potassium and chloride ions (Mount et al., 1999). Previous studies have shown that SLC12A7 is highly expressed in adrenal cancer (Brown et al., 2018; Brown et al., 2016), cervical cancer (Shen et al., 2000), ovarian cancer and breast cancer (Shen et al., 2001). We analyzed its expression in pan-cancer through TIMER public database and found that *SLC12A7* was overexpressed in many kinds of cancers, especially in gastric cancer. TCGA-STAD and GEO data also showed that SLC12A7 mRNA was significantly overexpressed in gastric cancer tissues. At the same time, ROC curve and survival analysis indicated that *SLC12A7* performed well in survival prediction. The high expression of *SLC12A7* was also indicated to be associated with poor prognosis in gastric cancer patients. The above analysis suggests that *SLC12A7* might act as an important factor in the process of gastric cancer evolution, which inspires us to further explore the molecular mechanism in occurrence and development of *SLC12A7* in gastric cancer.

We further validated the *SLC12A7* expression in paired cancer and adjacent non-tumor tissues. The result showed that *SLC12A7* was significantly highly expressed in gastric cancer tissues, consistent with the bioinformatic results. Our CCK-8 assay and Transwell experiments showed knockdown of *SLC12A7* could inhibit gastric cancer cell proliferation, migration and invasion ability. These results preliminarily confirmed that *SLC12A7* played a role in gastric cancer development but the way it exerted its effect needs further in-depth exploration.

*SLC12A7* knockdown also affected cell cycle progression and apoptosis. After *SLC12A7* silencing, the expression level of cell cycle-related protein CDK1 and CyclinA2 significantly reduced. Flow cytometry showed that the percentage of S-phase cells decreased while the percentage of G2-phase cells increased after *SLC12A7* knockdown. CDK1 is a key kinase that promotes G2/M transition and mitotic entry, its downregulation would impair this transition, induce G2 arrest, and consequently suppress cell cycle progression. Meanwhile, apoptosis detection showed that the percentages of early and late apoptotic cells significantly increased after *SLC12A7* knockdown, and the protein levels of apoptosis-related proteins cleaved-Caspase-3 and cleaved-Caspase-9 also significantly increased. This further proved that *SLC12A7* knockdown could inhibit cell cycle progression and induce apoptosis. Based on the above research results, it can be preliminarily inferred that *SLC12A7* may act as a tumor-promoting factor in the occurrence and development of gastric cancer.

EMT is closely associated with tumor-cell migration and invasion. In this study, *SLC12A7* knockdown increased E-cadherin expression and decreased vimentin expression in gastric cancer cells. These changes are consistent with attenuation of EMT-associated phenotypes and may partly explain the reduced migration and invasion observed after *SLC12A7* silencing. However, only two representative EMT markers were examined in our result, further analyses of additional EMT regulators and transcription factors are needed to clarify how SLC12A7 participates in EMT regulation.

The PI3K/AKT pathway is frequently dysregulated in cancer and participates in cell proliferation, survival, metabolism, and migration. Akt (protein kinase B) has been identified as one of the key kinases driving EMT (Wang et al., 2014; Lu et al., 2023). PI3K is a direct upstream kinase of AKT, whilst AKT is one of the primary downstream effectors of the PI3K signaling pathway (Liu et al., 2009; Vanhaesebroec et al., 2012). Phosphorylation of the p85 subunit at tyrosine 607 (Y607) mediates the activation of AKT by PI3K, particularly in the presence of conventional PI3K inhibitors (Han et al., 2018). In the present study, *SLC12A7* knockdown reduced the phosphorylation levels of PI3K and AKT without markedly altering their total protein levels. Ezrin re-expression partially restored PI3K/AKT phosphorylation in *SLC12A7*-deficient cells. These findings suggest that PI3K/AKT-related signaling may be associated with the phenotypic changes induced by *SLC12A7* modulation, and Ezrin is possibly involved in this adjustment process.

Ezrin is a membrane–cytoskeleton linker that connects membrane-associated proteins, including receptors, ion channels, and transporters, to the actin cytoskeleton (Kawaguchi and Asano, 2022). It also functions as a scaffold involved in the organization of membrane-associated signaling complexes. Previous studies have shown that Ezrin plays an important role in the activation of the PI3K/AKT pathway (Li N. et al., 2019; Zhang et al., 2025; Gautreau et al., 1999). Ezrin dysregulation has also been implicated in tumor-cell migration, invasion, survival (Song et al., 2020), and poor prognosis in gastric cancer (Zhu et al., 2024).

Our data suggest a potential functional relationship between SLC12A7 and Ezrin in GC cells. SLC12A7 expression was positively associated with Ezrin expression, and molecular docking predicted a favorable binding conformation between the two proteins. Immunofluorescence analysis further showed partial colocalization of SLC12A7 and Ezrin, mainly in the cytoplasm and perimembrane regions. Moreover, SLC12A7 knockdown reduced Ezrin, p-PI3K, and p-AKT levels, whereas Ezrin re-expression partially restored PI3K/AKT phosphorylation. These findings support a model in which Ezrin may participate in SLC12A7-associated PI3K/AKT signaling, although direct physical interaction and pathway causality require further validation.

The known transporter biology of *SLC12A7* may provide a possible explanation for its involvement in tumor-cell migration and invasion. *SLC12A7* is an electroneutral K^+^/Cl^−^ cotransporter involved in intracellular ion homeostasis, cell-volume regulation, and membrane homeostasis (Merc et al., 2000; Mount et al., 1999; Gamba, 2005; Arroyo et al., 2013). Previous studies in cervical, ovarian, breast, and adrenocortical cancers have shown that K^+^/Cl^−^ cotransporters, particularly *SLC12A7*, participate in tumor-cell proliferation, invasion, and membrane–cytoskeleton-associated migratory structures (Brown et al., 2018; Hsu et al., 2007a; Chen et al., 2009; Chen et al., 2010). Notably, in cervical cancer cells, *SLC12A7* has been reported to colocalize with Ezrin at lamellipodia and to contribute to cancer-cell invasion, suggesting that *SLC12A7* may function not only as an ion transporter but also as a membrane-associated regulator of cytoskeletal and signaling organization (Chen et al., 2009). These findings provide biological support for a potential link between *SLC12A7*-mediated ion and volume regulation and malignant phenotypes.

This study has several limitations that should be considered when interpreting the findings. Although our data suggest a link between *SLC12A7*, Ezrin, and PI3K/AKT signaling, they do not demonstrate a direct *SLC12A7*-Ezrin-PI3K/AKT cascade or confirm physical interaction between *SLC12A7* and Ezrin. Further validation using co-immunoprecipitation, pathway inhibition and rescue experiments, and broader analyses of EMT and PI3K/AKT downstream effectors will be necessary to establish causality. The clinical observations also remain correlative, as they are based mainly on bioinformatic analysis, tissue validation, and *in vitro* experiments; *in vivo* models are needed to determine whether *SLC12A7* regulates tumor growth, invasion, and metastasis under physiological conditions. In addition, K^+^/Cl^−^ cotransport activity, intracellular ion concentrations, cell volume, membrane tension, and pH-dependent responses were not directly assessed, leaving unresolved whether *SLC12A7* acts through its ion-transport function, non-transport-related signaling, or a scaffolding role.

## Conclusion

In conclusion, this study identified *SLC12A7* as a key gene associated with GC through integrated bioinformatic analysis and literature-based screening. qRT-PCR, Western blotting, immunohistochemistry, and immunofluorescence analyses confirmed the elevated expression of SLC12A7 in GC cells and tissues, whereas CCK-8 and Transwell assays demonstrated its functional association with malignant cellular phenotypes. Functionally, *SLC12A7* appears to promote GC-cell proliferation, migration, invasion, and cell-cycle progression while suppressing apoptosis. Clinically, high *SLC12A7* expression is associated with poor prognosis. Moreover, *SLC12A7* showed positive expression correlation and spatial colocalization with Ezrin, and the two proteins may cooperate in activating PI3K/AKT signaling. These findings provide a foundation for further investigation of the *SLC12A7*-Ezrin-PI3K/AKT signaling axis in GC progression and support *SLC12A7* as a potential therapeutic target.

## Data Availability

The original contributions presented in the study are included in the article/Supplementary Material, further inquiries can be directed to the corresponding authors.
