# Supplementary material for: SLC12A7 promotes malignant phenotypes of gastric cancer cells and is associated with Ezrin-related PI3K/AKT signaling
Source: Front Cell Dev Biol. 2026 Sep 15;14:1828288. doi: 10.3389/fcell.2026.1828288 (PMC13656039; doi:10.3389/fcell.2026.1828288)
Supplement: Supplementary file 1 [file Table1.docx]

Primer Sequence List

| Gene | Forward Primer（5' - 3'） | Reverse Primer（5' -3'） |
| --- | --- | --- |
| SLC12A7 | CGAGAGGACACTAATGATGGAG | TGAAGAGGTCTTTGAAACCAGA |
| GAPDH | GGAGCGAGATCCCTCCAAAAT | GGCTGTTGTCATACTTCTCATGG |
