# Supplementary material for: SLC12A7 promotes malignant phenotypes of gastric cancer cells and is associated with Ezrin-related PI3K/AKT signaling
Source: Front Cell Dev Biol. 2026 Sep 15;14:1828288. doi: 10.3389/fcell.2026.1828288 (PMC13656039; doi:10.3389/fcell.2026.1828288)
Supplement: Supplementary file 2 [file Table2.docx]

siRNA Sequences

| siRNA | Sense（5'-3'） | Anti-sense（5'-3'） |
| --- | --- | --- |
| si-NC | UUCUCCGAACGUGUCACGUTT | ACGUGACACGUUCGGAGAATT |
| si-SLC12A7#1 | GGUUGUCCAUAAAGAGGUUTT | AACCUCUUUAUGGACAACCTT |
| si-SLC12A7#2 | GGUGGAACAAGAGAGCUUCTT | GAAGCUCUCUUGUUCCACCTC |
| si-SLC12A7#3 | GCGUGGUCUUACGAGAUAATT | UUAUCUCGUAAGACCACGCTT |
